# Structured environments foster competitor coexistence by manipulating interspecies interfaces

**DOI:** 10.1371/journal.pcbi.1007762

**Published:** 2021-01-07

**Authors:** Tristan Ursell

**Affiliations:** 1 Institute of Molecular Biology, University of Oregon, Eugene, Oregon, United States of America; 2 Materials Science Institute, University of Oregon, Eugene, Oregon, United States of America; 3 Department of Physics, University of Oregon, Eugene, Oregon, United States of America; University of Illinois at Urbana-Champaign, UNITED STATES

## Abstract

Natural environments, like soils or the mammalian gut, frequently contain microbial consortia competing within a niche, wherein many species contain genetically encoded mechanisms of interspecies competition. Recent computational work suggests that physical structures in the environment can stabilize local competition between species that would otherwise be subject to competitive exclusion under isotropic conditions. Here we employ Lotka-Volterra models to show that interfacial competition localizes to physical structures, stabilizing competitive ecological networks of many species, even with significant differences in the strength of competitive interactions between species. Within a limited range of parameter space, we show that for stable communities the length-scale of physical structure inversely correlates with the width of the distribution of competitive fitness, such that physical environments with finer structure can sustain a broader spectrum of interspecific competition. These results highlight the potentially stabilizing effects of physical structure on microbial communities and lay groundwork for engineering structures that stabilize and/or select for diverse communities of ecological, medical, or industrial utility.

## Author summary

Natural environments often have many species competing for the same resources and frequently one species will out-compete others. This poses the fundamental question of how a diverse array of species can coexist in a resource-limited environment. Among other mechanisms, previous studies examined how interactions between species–like cooperation or predation–could lead to stable biodiversity. In this work we looked at this question from a different angle: we used computational models to examine the role that the environment itself might play in stabilizing species that compete with each other when in proximity. We modeled how species arrange themselves in space when the environment contains objects that alter the interfaces along which competing species meet. We found that these ‘structured’ environments can stabilize species coexistence, across a range of density of those objects and in a way that was robust to differing strengths of interspecies competition. Thus, in addition to biological factors and other forms of environmental variation, our work presents a potentially generic mechanism by which the physical structure of the environment can influence ecological outcomes and stabilize biodiversity.

## Introduction

Natural environments from scales of microbes [1–4] to large ecosystems [5–8] are replete with communities whose constituent species stably coexist at similar trophic levels, despite apparent competition for space and resources. In spatially limited ecosystems species grow until resources and/or interactions with other species (e.g. competition or predation) limit their populations, notably not necessarily at a constant level through time [9–11]. In some cases [12], the same set of species may exhibit qualitatively distinct relationships in a way that depends on available resources, with corresponding maintenance or loss of diversity. Species diversity and ecosystem stability have a complicated relationship [13,14], and qualitatively different theories have been developed to explain variations in species diversity and abundance in resource-limited natural environments [15,16]. At one extreme, the principle of competitive exclusion asserts that if more than one species is competing within a niche, variations in species reproduction rates resulting from adaptation to the niche will necessarily lead one species to dominate within that niche to the exclusion of all other species, potentially driving inferior competitors into other niches [17–21]. Thus, competition for resources within a niche would push ecosystems toward lower species diversity [22]. At the other extreme are so-called ‘neutral theories’ which offer the null-hypothesis that organisms coexisting at similar trophic levels are–*per capita*–reproducing, consuming, and migrating at similar rates, and hence maintenance of biodiversity is tantamount to a high-dimensional random-walk through abundance space [23–25]. Such models often require connections to an external meta-community to maintain long-term stability [26], lest random fluctuations will eventually drive finite systems toward lower diversity [27,28]. Many other mechanisms (which we cannot do justice to here) have also been proposed for maintenance of diversity in competitive ecosystems, including but not limited to: stochasticity and priority effects [29,30]; environmental variability [31]; models that encode specific relationships between species to maintain diversity [32] (including the classic rock-paper-scissors spatial game [11], cross-feeding [33–37], metabolic trade-offs [38–40], or cross-protection [41]); varied interaction models [42]; higher-order interactions–beyond pairwise–that stabilize diversity [43–46]; and systems where evolution and ecological competition happen simultaneously [47,48].

We do not take issue with any of these models/mechanisms, indeed, all of them are likely relevant and useful within certain contexts. Rather, the goal of this work was to determine ‘if’, and shed light on ‘how’, steric structures distributed in space impact long-term coexistence of locally interacting, mutually competing species in otherwise isotropic environments. We simulated Lotka-Volterra equations with spatial diffusion to demonstrate that physical, steric structure within an environment can be a robust mechanism for species coexistence, in contexts where both competition and dispersal are localized and resources are constant in space and time. A number of previous studies have used similar models to examine spatial heterogeneity [49,50]; some have examined founder and stochasticity effects [51], while others focus on temporal or spatial variability of biotic origin [52] (sometimes called ‘endogenous’ variability)–as opposed to the variability imposed by the environment (sometimes called ‘exogenous’ variability). For continuum Lotka-Volterra models the number of distinct pairwise interactions between species grows quadratically, and hence theoretical work tends to focus on interactions between two or three competitor species. Previous work speculated that “the classical theory for coexistence generated by the well-mixed assumption behind the Lotka-Volterra competition equations may not be sufficient to generate coexistence when ecological interactions and dispersal are localized in space”[52]. While the definition of ‘localized in space’ has some flexibility, to our reading the literature does not systematically address how coexistence depends on the spatial scale of steric structure, disorder/spatial arrangement of structures, and distributions of competitive parameters across many species–we break ground on all of these fronts in this work, albeit within a necessarily limited range of the available parameter space.

Microbial ecosystems present a particularly attractive test-bed for these ecological ideas. From a practical point of view, they are small and fast growing, relatively easy to genetically manipulate, and can be grown in controlled and customizable synthetic environments [35,53–56], such as microfluidics [57,58]. Characterizing the forces and principles that establish and maintain microbial diversity is of significant interest in health-relevant settings like the human gut [59–61] and in the myriad contexts where soil microbiota impact natural or agricultural ecosystems [1,62]. These contexts motivate the model herein discussed, which can be conceptualized as a locally interacting, multi-species microbial ecosystem whose only spatial anisotropy is the presence of steric structure–resources are spatially and temporally isotropic–and the only form of organismal motility is isotropic diffusion. Further, the constituent species are assumed to have pairwise competitive relationships that are constant in time and space. We adhered to reasonable simplifications that make computations tractable, and then focused on the role that physical structure of the environment plays in long-term dynamics of such *in silico* ecosystems. Within the context of the model and its assumptions, our results were clear–steric structures distributed throughout the environment foster coexistence in a way that depends less on the specific arrangement of those structures and more on the length scale of separation between them [63]. This structural stabilization was also robust to significant differences in the strength of competitive interactions between species, and the degree of stabilization positively correlated with decreasing structural scale. Finally, within the context of the model, we provide evidence that the stabilizing effects of steric structure extend to many species in competition with each other, subject to constraints on available space.

## Results

### Competition and structural model

We modeled intra- and inter-species interactions using a canonical spatial Lotka-Volterra (LV) framework, with simplifying assumptions motivated by attributes of microbial ecosystems. For all species, times, and locations we assume that the carrying capacity per unit area of the environment is the same, that the basal growth rate *r* is the same, and that organismal migration can be described by random walks with the same diffusion coefficient *D*. Using those assumptions, the system of partial differential equations (PDEs) that describe an *N*-species spatial LV model can be non-dimensionalized and, without loss of generality, written as

$$\frac{\partial S_{i}}{\partial t}=\nabla^{2}S_{i}+S_{i}\left(1-\sum_{k=1}^{N}S_{k}(1+\frac{1-\delta_{ik}}{P_{ik}})\right).$$ (1)

Each focal species *S*_*i*_ has a local concentration from zero to one in units of the carrying capacity, time is measured in units of inverse growth rate *r*^-1^ and the natural length scale $\lambda =\sqrt{D/r}$ is proportional to the root mean squared distance an organism will move over a single doubling time. Thus, the results do not depend on choice of *r*, *D*, or carrying capacity, rather those parameters set the scales of the simulation. Self-interactions and simple competition for space are accounted for by the constrained carrying capacity and the corresponding sum over *S*_*k*_, and thus the diagonal elements of the interaction matrix, *P*_*ii*_, are removed by the Kronecker delta, *δ*_*ik*_. Pairwise interactions between species are described by the off-diagonal matrix elements *P*_*ik*_ which are the local concentrations of species *S*_*k*_ above which *S*_*k*_ actively reduces the local concentration of *S*_*i*_. For a given pair of species that form a competitive interface, if *P*_*ik*_ > 1 then the local concentration of *S*_*k*_ needed to *reduce* the local concentration of *S*_*i*_ is greater than the carrying capacity, and hence *S*_*k*_ at most reduces the effective local growth rate of *S*_*i*_, not its local abundance. As *P*_*ik*_ increases, the strength of interaction decreases. If 0 < *P*_*ik*_ < 1 then there is some concentration of *S*_*k*_ below the carrying capacity (*P*_*ik*_ < *S*_*k*_ < 1)that reduces the abundance of *S*_*i*_, which we refer to here as ‘active’ competition. For any two species *i* and *k* that are adjacent in space with 0 < *P*_*ik*_ < 1 and 0 < *P*_*ki*_ < 1, a (potentially) mobile competitive interface will form between those species, regardless of steric structure. In this work, we focus on situations with 0 < *P*_*ik*_ < 1 for all *i* and *k*, as that is the parameter sub-space (in this model) that tends to result in competitive exclusion and reduced species coexistence in the long-time limit–we refer to this subset of models as ‘all-to-all’ (ATA) competition. This subset of models admits stationary, long-time solutions that correspond to competitive exclusion, where the number of final species is less than that number of initial species. It also admits competitive hierarchies and alliances–like the three-species rock-paper-scissors game and its variants (which we do not explicitly explore here) whose solutions can, for instance, adopt limit-cycle or chaotic dynamics [64] that maintain coexistence for long periods of time without steric structure.

Neglecting intrinsic permutation symmetries, the *N*(*N* − 1) pairwise interaction parameters for each *in silico* ecosystem can be thought of as a directed graph whose edges connect each pair of species. As *N* increases, the dimensionality of the parameter space grows quadratically, precluding exhaustive computational characterization of this model. Thus, we restricted our simulations and analysis to a single decade in species number 2 ≤ *N* ≤ 12, primarily focusing on *N* = 8 (just above the mean of that range). Previous work [64] more extensively examined the same model for *N* = 2. For each *in silico* ecosystem (i.e. over all replicates) we sampled a uniform random distribution of values for the off-diagonal elements *P*_*ik*_ whose mean was ⟨*P*⟩ = 0.25 and whose width was specified by the parameter Δ*P*, subject to Δ*P*/2 < ⟨*P*⟩ so that all interaction parameters remained competitive. In total, we sparsely sampled a small fraction of the 0 < *P*_*ik*_ < 1 parameter space (that fraction being proportional to Δ*P*^*N*(*N*−1)^). The value ⟨*P*⟩ = 0.25 indicates that on average when the local concentration of a given species reaches one-quarter of the carrying capacity, active reduction of neighboring competitors will occur. Distribution width Δ*P* sets the maximum magnitude of competitive asymmetry between any two species. We explored Δ*P* over a limited range corresponding to 0.175 ≤ *P*_*ik*_ ≤ 0.325, or, in terms of concentration, where active competition could (depending on sampling) set-in between ~1/6 and ~1/3 of the carry capacity. These parameters were chosen because the attendant simulations displayed the full range of possible number of coexisting species in the long-time limit, for *N* = 8). For each value of Δ*P* and structural parameters, we performed 30 to 50 simulations with a unique random sampling of the interaction parameters *P*_*ik*_. All simulations had initial conditions in which every grid position had a low (0.2%) probability of being populated by any one of the available species, such that each species could grow and claim territory before locally competing.

Our *in silico* environments were square 2D planes with steric pillars distributed in the simulation space. Both the pillars and the bounding box were modeled with reflecting boundary conditions, thus, like a grain in soil or tissue in a gut, these pillars do not allow free transport through them, nor microbes to occupy them. In a structured environment, the shape and movement of interspecies boundaries are primarily impacted by steric spacing [64], and thus for simplicity the radii of the pillars were held fixed at *R* = 3 in dimensionless units for all simulations. We varied the mean distance between steric objects relative to pillar radius (Δ*x*/*R*), which we refer to as the ‘structural scale’, and we varied the degree of disorder in the arrangement of those steric objects. Disorder was introduced by translating each pillar in a random direction by an amount drawn from a uniform random distribution whose width is reported relative to the structural scale. Thus, disorder is characterized by a continuous dimensionless variable *δ*, which when equal to zero means the pillars are arranged in an ordered triangular lattice, and as *δ* increases the pillars approach a random arrangement in the simulation space (including the possibility of overlap) (see S1 Fig).

This model is appropriate for describing locally competitive interactions in which the movement of organisms is described by a random walk and where the mechanisms underlying competition occur between nearby cells/species. Examples of such ecosystems include situations where microbial species compete for the same constant and isotropically distributed pool of resources, and actively reduce competitor abundances through (e.g.) Type VI secretion system mediated killing [65,66], secretion of toxins [67,68] or antibiotic antagonism [69,70]. Analysis of bacterial genomes indicates that (at least) a quarter of all sequenced species contain loci encoding for mechanisms of active competition toward other species [71] (though not necessarily for the purposes of consuming them as prey). Additional PDEs would be required to describe: (i) highly motile cells whose movements are (e.g.) super-diffusive, (ii) chemotaxis in exogenous chemical gradients, (iii) flow/advection, or (iv) the production, potency, transport and decay of secreted toxins that diffuse faster than organismal diffusion. This system of PDEs (which is not new [72,73]) represents a baseline set of assumptions and corresponding phenomena from which to build more complex models [74] of structured and locally competitive population dynamics. In particular, it is part of a larger class of boundary-forming models that localize competitive interactions to zones between competitors, and thus it is a reasonable starting point for understanding the effects of steric structure in such contexts.

Finally, inherent in this modeling framework it is worth noting additional limitations and simplifications. These systems of PDEs are deterministic, that is, with the same interaction parameters, structural parameters, simulation size and initial conditions they produce the exact same dynamics. Stochasticity enters our simulations through the random initial conditions, disorder, and random samplings of the interaction parameters. A number of excellent studies have examined low*-N* systems using fully stochastic dynamics [75–79], revealing quantitative differences between deterministic and stochastic models, as well as qualitative differences over long time scales where stochastic fluctuations can drive a system into new parts of phase space, for example, into extinction cascades [80,81] or mobility-dependent coexistence [75,77,82]. Similarly, recent work [83] examined how non-diffusive mobility (i.e. power-law jump distributions that produce random long-range dispersal) spatially structures genotypes in an environment, finding that certain parameter regimes produce domain structures similar to those seen in this work, while other regimes produce more complex dynamics. Such non-local dispersal mechanisms would, almost certainly, alter the qualitative conclusions presented here because non-local jumps ‘bypass’ the limitations in organismal mobility imposed by structured interfaces and local diffusion.

### Environmental structure stabilizes local all-to-all competition

**Fig 1:**
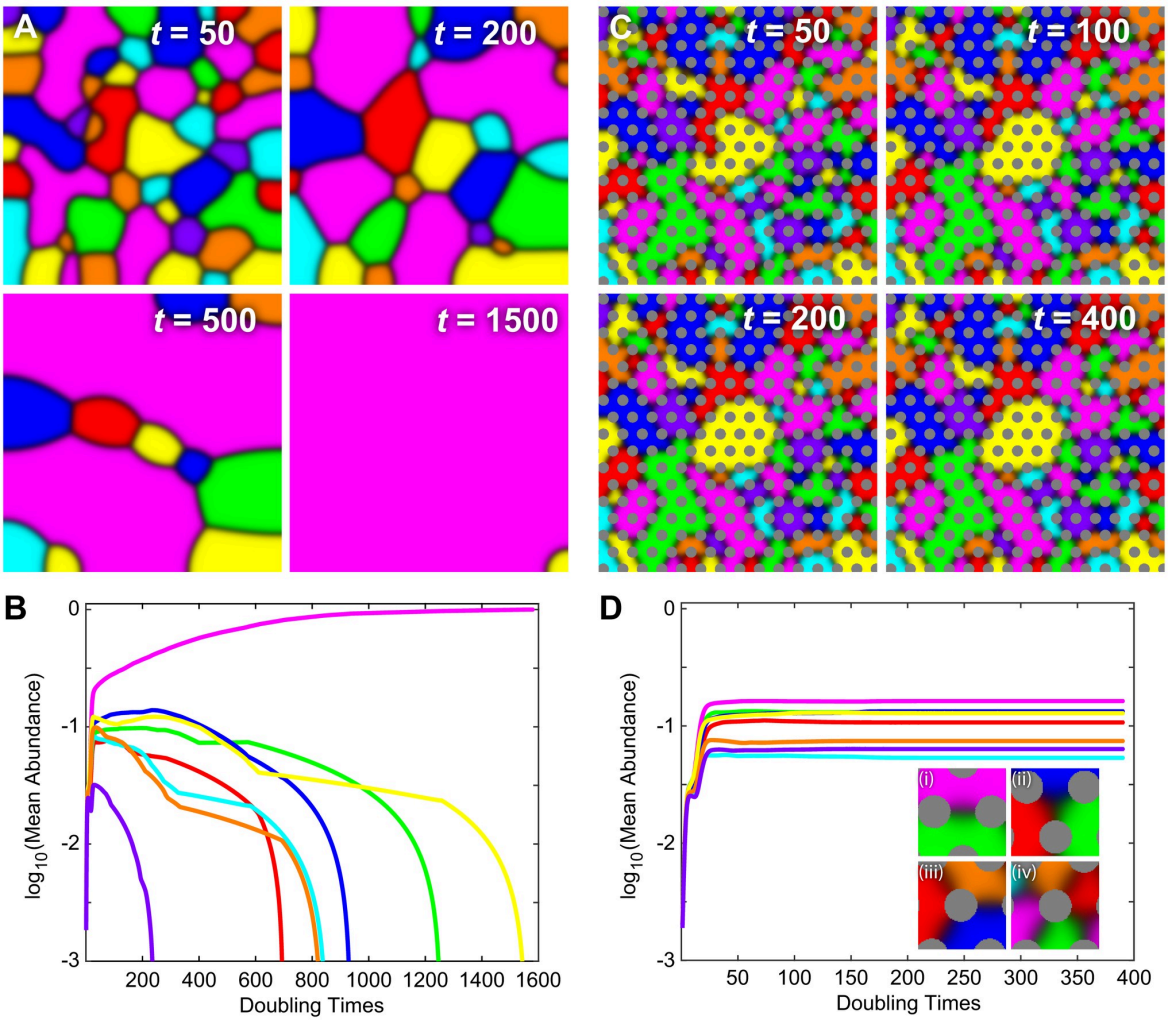
Steric structures stabilize an *in silico* multi-species ecosystem. Shown here are two 8-species *in silico* ecosystems in which all species are actively competing with all other species and the competition parameters are equal for all pairwise interactions. **(A & B)** Simulation of competition in an isotropic environment. If initial species representation is statistically equal, each species has equal probability of dominating the environment in the long-time limit. However, in any single simulation the dynamics of interspecies boundaries lead to a single dominant competitor in the long-time limit. **(C & D)** When species compete in an environment with ordered steric structures (*R* = 3, Δ*x*/*R* = 3.5, and *δ* = 0), interspecies boundaries that are mobile under isotropic conditions quickly ‘pin’ between steric objects and arrest the genetic-phase coarsening that leads to a single dominant competitor, thereby robustly producing stable representation of all species. Steric structures also permit situations where 3 or more species form a ‘junction’ around a steric object (Diii and Div). In both simulations *L* = 150, ⟨*P*⟩ = 0.25, and Δ*P* = 0.

As a computational proof of principle–that steric structure can impact coexistence of locally competing species–we compared the spatial population dynamics of an 8-species system with and without the inclusion of steric structure. In both cases, each pair of species that shared an interface engaged in active (population reducing) competition, each characterized by a pair of off-diagonal matrix elements (56 parameters in total). In Fig 1 we show the simplest version of this comparison, where the strength of the competitive interaction is equal between all pairs of species (i.e. all off-diagonal elements have the same value, Δ*P* = 0).

Even in this state of balanced competition, if a system lacks steric structure, and is otherwise isotropic, a single dominant competitor will emerge to the exclusion of all other species given enough time [84] (Fig 1A and 1B). Other salient dynamics emerge from these PDEs and assumptions as well. Spatial domains of each species are determined by an interplay between the curvature of the interface between any two species and the relative values of the competition parameters for the species that meet at that interface [64]. Neglecting, for the moment, spatial junctions between three or more species, all competition interfaces in this model are, by geometric necessity, two species interfaces–thus analysis of two-species interfaces generally informs analysis for systems with *N* > 2. These model-specific observations are similar to dynamics seen in Potts models [85–87] (a generalized version of the Ising model) that can display multiple, mutually coarsening phases. If competition at a particular two-species interface is balanced (i.e. *P*_*ik*_ = *P*_*ki*_) then interfacial curvature (linear curvature in 2D space) is the only determinant of interface movement; straight interfaces do not translate and curved interfaces translate in directions that minimize the interface length (akin to a ‘surface tension’ between competing species, whose dimensionality depends on the spatial dimensions of the system). Notably, these interfacial dynamics lead to competitive exclusion; domains of any competitor that are enclosed by equal or stronger competitors will, in open space, decrease in size until local extinction (thereby reducing interface length). System dynamics become more complex in the case that interactions at a two-species interface are unbalanced (i.e. *P*_*ik*_ ≠ *P*_*ki*_). Straight interfaces translate in the direction of the weaker competitor, and below a critical interfacial curvature

$$\kappa_{ik}^{\left(P\right)}\propto \frac{1}{\lambda }\frac{\left|P_{ik}-P_{ki}\right|}{P_{ik}+P_{ki}},$$ (2)

stronger competitors will invade the space of a weaker competitor–ultimately to the exclusion of the weaker competitor–even though the interfacial geometry gives the weaker competitor a local concentration advantage [64]. In the statistical ensemble of sampled interaction parameters

$$\langle \kappa^{\left(P\right)}\rangle ≃\frac{1}{2\lambda }\frac{\Delta P}{\langle P\rangle }$$ (3)

for small Δ*P*/⟨*P*⟩. Excepting literal edge cases, wherein boundaries between species contact multiple parallel edges of the simulation space, the dominance of a single competitor in isotropic space is robust to changes in the size of the simulation space, the number of species and the values of interaction parameters, given enough time and assuming the system does not exhibit dynamic coexistence (e.g. as in rock-paper-scissor systems). Further, due to founding effects and depending on the specific initial conditions, the dominant competitor is not necessarily the competitor whose interaction parameters are the smallest (i.e. most lethal to other species). For instance, if a weaker competitor encompasses the domain of a stronger competitor with an interface whose curvature is everywhere greater than $\kappa_{ik}^{\left(P\right)}$, the weaker competitor will locally exclude the stronger competitor.

In contrast to robust competitive exclusion in isotropic space, the inclusion of physical structure leads to long-term, stable representation of multiple (and often all) species across a range of structural scales and interaction parameters. In Fig 1C and 1D we show the evolution of balanced competitive interactions between 8 species with the same initial conditions and interaction parameters as Fig 1A and 1B, but in the presence of a triangular lattice of steric pillars. In this system, the abundances of all 8 species rapidly equilibrated leading to stable coexistence. In this stable state, each species established spatial domains with boundaries primarily composed of two-species interfaces whose movement has the same relationship to interaction parameters and interfacial curvature as described above (Fig 1Di). However, inclusion of steric structure adds additional boundary conditions that locally slow or potentially halt interface dynamics, thus fostering coexistence. The steric pillars also stabilized a number of distinct ‘junctions’ between species, including three-way junctions in open space (Fig 1Dii), three-way junctions centered on a pillar (Fig 1Diii), and four-way junctions centered on a pillar (Fig 1Div). Isotropic systems without steric structure can transiently support two-species interfaces and free three-way junctions (Fig 1A), but stable three- and four-way junctions must be associated with steric objects in the local environment. Junctions centered on pillars can support more than four species if pillars are large in comparison to the thickness (*w*) of the competition interface (i.e. if 2*πR*/*N* > *w*). Though, such junctions did not occur in our simulations with random initial conditions–we only observed these higher-species junctions under contrived conditions (see S2 Fig).

### Stable coexistence is robust to fluctuations in steric structure and competition asymmetries

**Fig 2:**
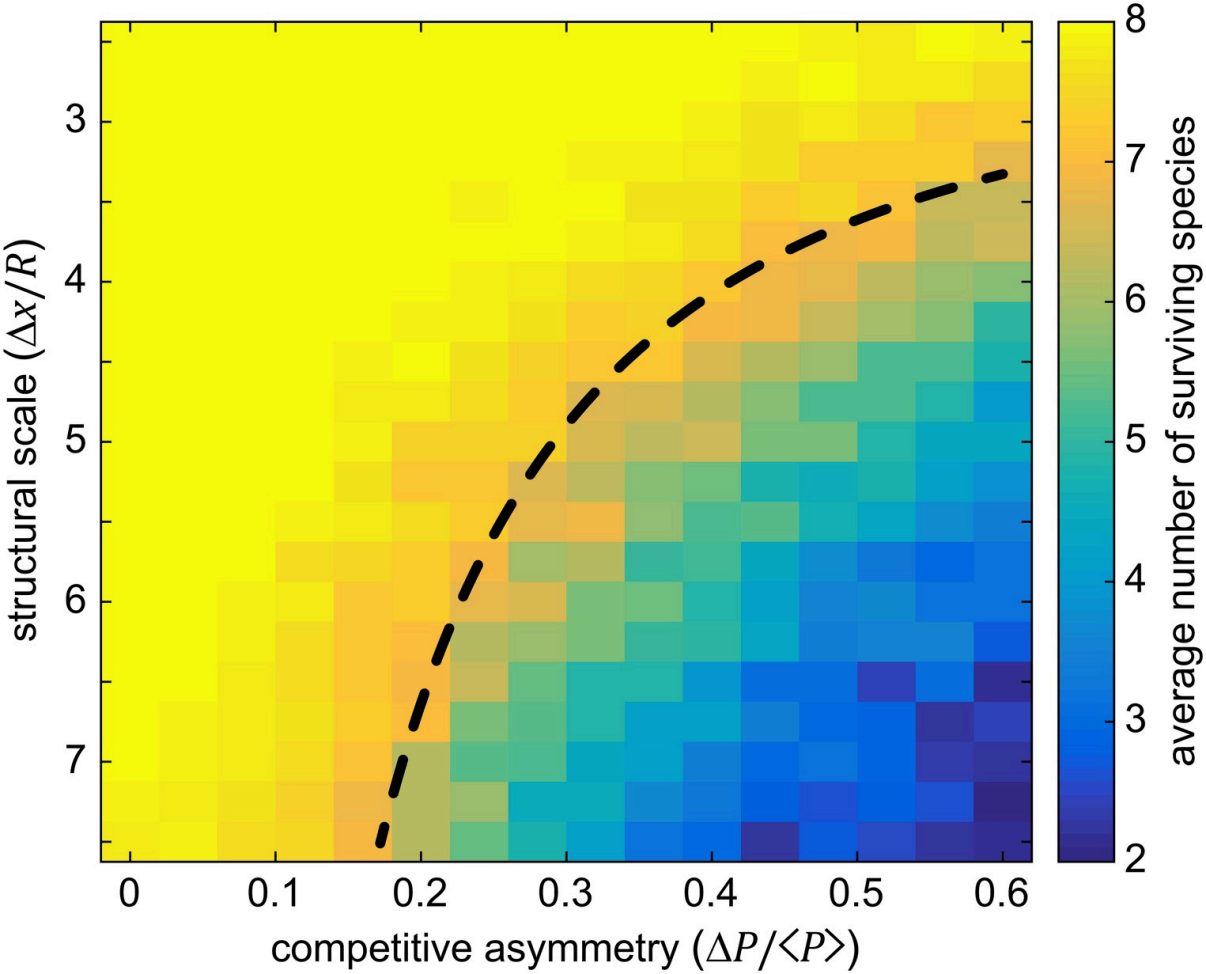
The number of species an environment can stably support depends on the degree of competition asymmetry and the structural scale. Simulations were performed across a range of competitive asymmetries characterized by the dimensionless parameter Δ*P*/⟨*P*⟩ and across a range of structural scales characterized by the dimensionless parameter Δ*x*/*R*. Each pixel corresponds to the mean of 30 simulations each with a unique random sampling of initial conditions (as described in Methods), a unique random sampling of the interaction matrix elements using Δ*P* and ⟨*P*⟩, and a fixed structural scale. Structural scale and competitive asymmetry were both potent modulators of species coexistence, with smaller structural scale and lower competitive asymmetries leading to stable representation of all species (yellow region). The black dashed line is a zero fit-parameter model relating the competitive asymmetry to the maximum curvature possible for a given structural scale, showing that relatively simple geometric considerations capture the onset of species loss. In all simulations *L* = 150, ⟨*P*⟩ = 0.25, *R* = 3, and *δ* = 0.

Next we held the degree of structural disorder fixed at zero (*δ* = 0) and explored how changes in the structural scale (Δ*x*/*R*) affected the number of stably coexisting species. Along one dimension, we held ⟨*P*⟩ = 0.25 and varied the interaction parameter Δ*P*, subject to the constraint that Δ*P*/2 < ⟨*P*⟩. Along a second parametric dimension, we varied the structural scale while holding pillar radii fixed. In Fig 2 we measured the mean number of species coexisting in the long-time limit as a function of both the width of the interaction-parameter distribution and the structural scale, with 30 replicates per parameter set for a total ~11,000 simulations. Consistent with previous work on two-species systems [64], the average number of coexisting species declined sharply both as competition asymmetry increased (i.e. as Δ*P*/⟨*P*⟩ increased) and as the structural scale increased. Conversely, systems with smaller structural scales and/or smaller competitive asymmetries robustly retained all eight species in the long-time limit (solid yellow region in Fig 2).

Assuming that competition interfaces meet reflecting boundaries at right angles and form circular arcs, a straightforward geometric calculation [64] shows that the maximum interface curvature between two pillars with radius *R* and center-to-center separation Δ*x* is given by

$$\kappa_{c}^{\left(struc\right)}=\frac{1}{R\sqrt{{\left(\Delta x/2R\right)}^{2}-1}}$$ (4)

Thus, setting the two critical curvatures equal to each other ($\langle \kappa_{c}^{\left(P\right)}\rangle =\kappa_{c}^{\left(struc\right)}$), we find a relationship that approximates the boundary between the regime of possible stable coexistence of all species and a regime with necessarily reduced species coexistence

$${\left(\frac{\Delta x}{R}\right)}_{crit}=2\sqrt{1+{\left(\frac{2\lambda }{R}\frac{\langle P\rangle }{\Delta P}\right)}^{2}}.$$ (5)

This regime boundary is shown overlaid on Fig 2, and applies to the specific statistical and structural context used here, though likely similar relationships exist for other distributions of interaction parameters and steric shapes. The equation indicates that for specified length scales, a given ensemble of interaction parameters defined by Δ*P*/⟨*P*⟩, and regardless of the carrying capacity, there is a critical structural scale (in a pillared environment) below which all species *can* coexist. Said differently, the equation indicates that smaller structural scales can support a wider range of interaction parameters among coexisting species, or conversely that larger structural scales select for stronger competitors. The accuracy of this approximation improves as the pillar radius becomes large compared to the width of the competitive interface.

**Fig 3:**
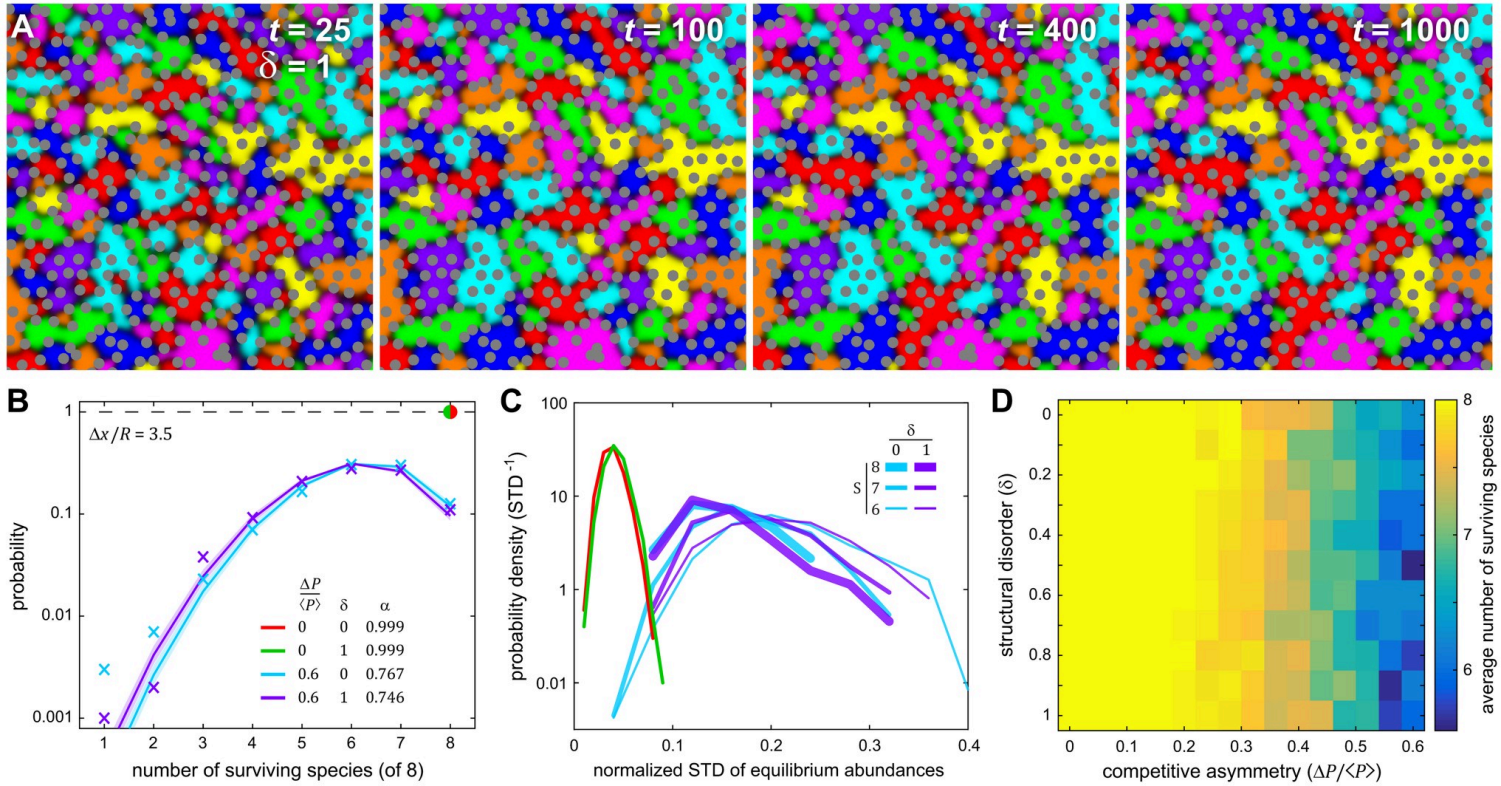
Species coexistence is robust in the presence of structural disorder. **(A)** Similar to Fig 1C, these panes show the time evolution of an 8-species system where steric pillars were randomly perturbed from a triangular lattice (*δ* = 1). From random initial conditions the system rapidly equilibrated to a stable configuration that supported all 8 species. **(B)** Simulations were performed to measure the probability distribution for the number of long-term coexisting species under four conditions (1,000 each): high and low competitive asymmetry and high and low structural disorder. The structural scale was held fixed. Without competitive asymmetry all species survived in all simulations, with or without disorder (red/green dot). With high competitive asymmetry, the probability distributions spread across all possible numbers of species with relatively little distinction between ordered and disordered systems (colored X’s). Those distributions were well-described by a modified binomial distribution (solid lines) with an ensemble-average single-species survival probability of *α* ~ 0.75. The distributions exhibit larger variation where there are less data (i.e. lower probabilities, from one to three surviving species). **(C)** For each simulation, the normalized standard deviation (NSD) in equilibrium abundances was measured, and those values are shown here as a histogram for each of the four conditions. In the absence of competitive asymmetry (green, red), the mean NSD was low (~0.05 on maximum scale of 1). When competitive asymmetry was high, we examined the NSD for all simulations that had 6, 7, or 8 surviving species (cyan, purple). All of those distributions were significantly wider and had significantly higher mean NSD’s, meaning that competitive asymmetry increases the variation in species abundance at equilibrium, regardless of how many species stably coexist. **(D)** We compared the average number of surviving species across a range of competitive asymmetry and disorder (0 ≤ *δ* ≤ 1). Those distributions showed little variation with disorder and, as a function of competitive asymmetry, had a mean pairwise correlation of 0.97 (see S4 Fig). Thus our data suggest that structural scale has a stronger effect than disorder (at least in this portion of parameter space). In all simulations *L* = 150, ⟨*P*⟩ = 0.25, and *R* = 3.

While these results are supportive of the stabilizing effect of steric structure on long-term species coexistence, rarely do natural environments contain highly ordered (*δ* = 0) steric structures, thus we explored how disorder affects species abundance at a fixed structural scale. First, we simulated the simpler case where all eight species had balanced competitive interactions (like Fig 1) and examined the population dynamics in the presence of disordered steric structures (*δ* = 1). Like the ordered case, disordered systems with balanced competitive interactions displayed stable representation of all eight species (Fig 3A). We then compared the probability distribution for the number of coexisting species in the long-time limit across four conditions (1,000 simulations for each): with and without competitive asymmetry, and with and without structural disorder, as shown in Fig 3B. When Δ*P*/⟨*P*⟩ = 0 the number of species remained at the maximum value across all 1,000 simulations whether or not the steric structures were disordered. When competitive asymmetry was introduced the probability distribution for the number of stably coexisting species expanded across all possible values (1 to 8) and peaked between 6 and 7 species, regardless of whether the system was ordered or disordered. Those distributions were quantitatively similar, indicating that disorder was not a strong determinant of stable species coexistence.

Across each of those same four conditions, we examined the probability distributions that describe the number of coexisting species in the long-time limit and applied the simplest rule that emerges from the statistical ensemble of initial conditions, competitive asymmetries, and disorder. For a given set of conditions we assumed that there is some probability *α* that a species randomly selected from the full ensemble of simulations will survive in the long-term. This corresponds to a binomial distribution whose normalization is modified to account for the fact that there is no chance that all *N* species will die

$$p_{n}(N,\alpha )=\frac{N!\alpha^{n}{\left(1-\alpha \right)}^{N-n}}{n!(N-n)!(1-{\left(1-\alpha \right)}^{N})},$$ (6)

where *N* is the maximum (initial) number of species and *p*_*n*_ is the probability of 1 ≤ *n* ≤ *N* species coexisting at equilibrium. We used maximum-likelihood estimation to fit this model to the survival distributions and thus determined *α*, whose value (0 < *α* < 1) corresponds to the ensemble average probability that any single species survives in equilibrium within a randomly assembled ecosystem. This value depends on the initial number of species *N*, simulation size *L*, structural parameters Δ*x* and *R*, the disorder *δ*, and sampling parameters ⟨*P*⟩ and Δ*P*; the exact functional dependence between those parameters and *α* is not yet clear. This approximation for *p*_*n*_ captures the bulk of the survival distribution and mis-estimates the occurrence of rare events compared to the data. The fit values suggest that smaller systems (lower *L*) and systems with higher competitive asymmetries both have lower per-capita survival probabilities (*α*). For instance, examining systems with higher competitive asymmetry, we found that larger systems (*L* = 150) with or without disorder had quantitatively similar survival probabilities– $\alpha =0.746_{-0.010}^{+0.009}$ and $\alpha =0.767_{-0.009}^{+0.009}$ respectively–whereas under the same conditions a smaller system (*L* = 75) had survival probabilities of $\alpha =0.600_{-0.011}^{+0.011}$ and $\alpha =0.626_{-0.011}^{+0.011}$, respectively (see S3 Fig).

We used those same 4,000 simulations to assess the variability in species abundances resulting from the four conditions. At equilibrium, each coexisting species occupied a fraction (between 0 and 1) of the available simulation area. For each replicate we measured the standard deviation of that fraction across all coexisting species. We then repeated that for all replicates under each of the four conditions, to generate a distribution of normalized standard deviations (NSD) in species abundance for each condition. NSD values closer to zero indicate that all coexisting species within a replicate have roughly the same abundance, whereas values approaching one indicate that abundance variations between species are comparable to the size of the system itself. In the case of balanced competitive interactions, the probability distributions for abundance variations were nearly identical for the ordered and disordered systems, and the mean NSD was low (~0.05, see Fig 3C), meaning that if competitive interactions are balanced all species are have roughly the same abundance. However, introducing moderate competitive asymmetry meant that some species intrude into the territory of other species, leading both to lower species diversity and to larger variations in the abundance of surviving species. As such, we report the distribution of NSDs for all simulations that had equilibrium species numbers of either *S* = 6, 7 or 8 (Fig 3C). Again, disorder had little effect on those probability distributions. Systems that experienced extinctions of zero (*S* = 8), one (*S* = 7) or two (*S* = 6) species had higher mean NSDs (by a factor of 3 to 5) and wider distributions of NSDs as compared to balanced competition, with lower equilibrium values of *S* corresponding to larger mean variations in abundance. Additionally, we performed a wider sampling of the degree of disorder and the competitive asymmetry, and found that the average number of stably coexisting species showed little dependence on the degree of structural disorder (Fig 3D). When we correlated the mean number of stably coexisting species as a function of the competitive asymmetry across all values of *δ*, the average correlation coefficient was 0.97 (see S4 Fig), meaning that the relationship between average number of coexisting species and competitive asymmetry showed little dependence on disorder in the range 0 ≤ *δ* ≤ 1.

As a final characterization of spatial structure, we examined the density with which interspecies boundaries connected steric objects. Ignoring pillars at the edge of the simulation space, every steric object has a set of Voronoi nearest neighbors, typically 5 to 7 in disordered systems and exactly 6 in a triangular lattice (see S5 Fig). Across our simulations, the vast majority (~ 98%) of all interspecies boundaries connected pillars that were Voronoi nearest neighbors, which is expected given geometric constraints. The number of those connections per unit area relative to the total number of possible Voronoi connections per unit area is a dimensionless measure of the amount of localized competition in a physically structured system–below we refer to this as the ‘connection density’, whose values lie between 0 and 1. When examined through that lens, disorder, at least for balanced interactions, had a significant effect on the distribution of connection densities across the ensemble of simulations, with ordered systems exhibiting higher connection densities (see S5 Fig). However, when examined under moderate levels of competitive asymmetry connection density significantly decreased (consistent with abundance variation increasing) and the difference between ordered and disordered systems again became small. These data suggest that in sterically structured systems higher levels of competitive asymmetry, somewhat counterintuitively, correlate with less competition as measured by the density of competitive interfaces in the system, because boundaries between mismatched competitors are less stable.

### Structure stabilizes larger numbers of competitors with system-size dependence

All of the simulations discussed up to this point were performed within the same size grid *L* = 150 (with the exception of S3 Fig, *L* = 75). Under any set of parametric conditions that lie within the assumptions of our model, the absolute minimum domain size for a given species is set by the area of a triangle formed by three pillars that are all mutual Voronoi neighbors (so-called ‘Delaunay triangles’[88]). Thus a system that is not large enough to contain domains of at least that size for each of *N* unique species cannot support all *N* species–this establishes a minimum system size for a particular number of species that scales as *N*(Δ*x*)^2^; all of our simulations were well above this strict limit. However, disordered systems have an additional system-size dependence. All else being equal, as the system size grows the probability distribution for the equilibrium number of species (e.g. Fig 3B) shifts toward the maximum number of species (i.e. *α* → 1). The mechanism behind this shift is that as the system increases in size, there are more opportunities for randomly positioned steric objects to create a zone in which a weaker competitor is enclosed by an effectively smaller structural scale. We confirmed this by measuring the system-size independent distribution of local structural scale (S6 Fig). In an ordered system, the distribution of local structural scale is a delta-function centered on the lattice constant–all nearest-neighbors pillars are the same distance from each other. As disorder increased we found that Voronoi zones (i.e. potential species domains) emerged whose maximum convex edge-length was smaller than the mean spacing between pillars. Thus, the maximum interface curvature that can exist between two species in such domains is greater than in ordered systems with the same mean spacing. In such zones a species whose interaction parameters render it too weak to compete in an ordered system, could potentially survive. The average number of these zones per unit area is system-size independent, thus increasing system size linearly increases the average number of those zones, and thus the per-capita survival probability *α* increases (e.g. compare Fig 3B and S3 Fig), as does the survival probability of weaker competitors.

**Fig 4:**
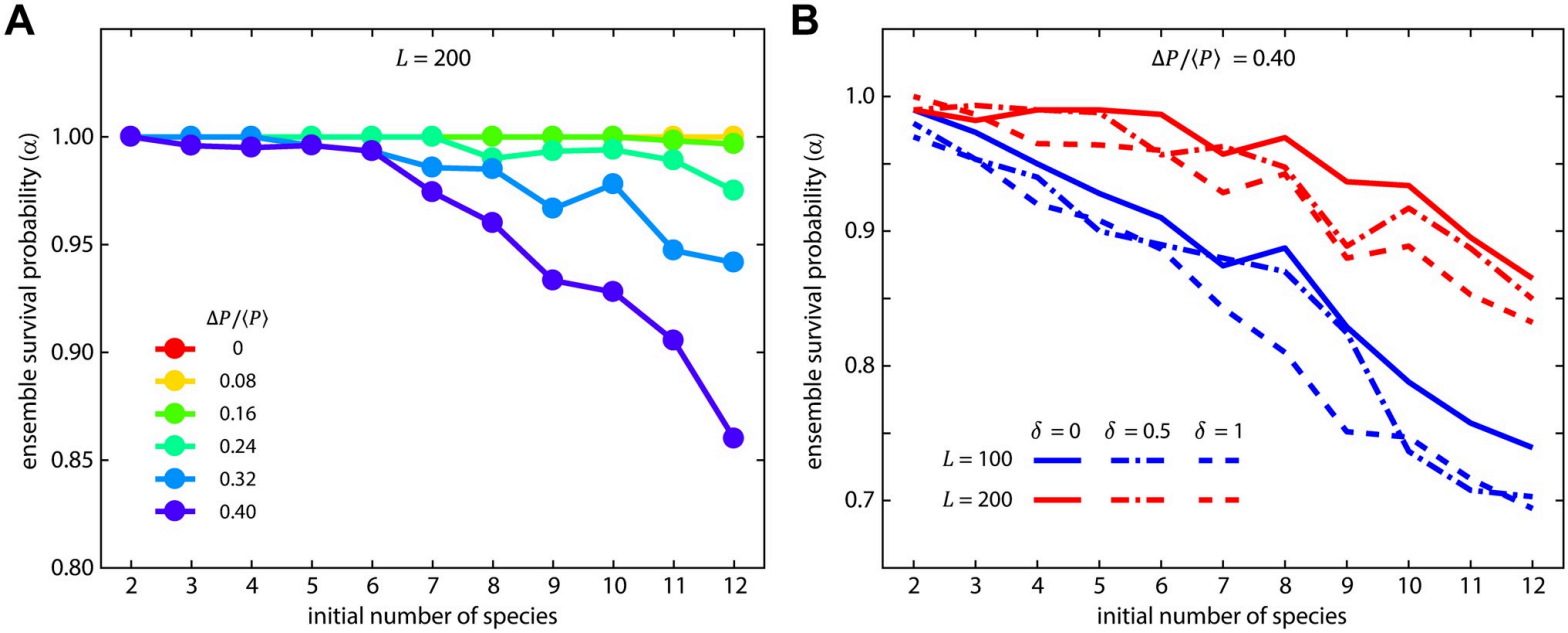
Structure stabilizes larger numbers of species, but increasing competitive asymmetry increases species loss. **(A)** Holding the structural scale fixed with no disorder, we measured the survival probability as a function of the initial number of species, between 2 and 12, across multiple values of competitive asymmetry. For lower values of competitive asymmetries, the final and initial numbers of species were essentially equal (α ~ 1). Higher levels of competitive asymmetry resulted in increasing degrees of species loss as the number of initial species increased. This amplification of species loss is related, at least in part, to the same interplay between structural scale and maximum interface curvature that caused species loss in Fig 2. **(B)** Comparing identical conditions between two different system sizes (*L* = 100 and *L* = 200), the effects of disorder are relatively small in comparison to the effects of system size, with smaller system sizes (blue lines) showing a significant amplification of reduction in survival probability as compared to the larger system. In all simulations ⟨*P*⟩ = 0.25 and *R* = 3.

Finally, in an ordered system we explored how the ensemble survival probability depended on the initial number of species, across the range 2 ≤ *N* ≤ 12, for two system sizes and multiple competitive asymmetries. First, we measured the ensemble survival probability (*α*) as a function of both competitive asymmetry and the initial number of species, with 50 replicates for each parameter set (Fig 4A). For low competitive asymmetry across all values of *N*, the ensemble survival probability was approximately 1, meaning all species coexisted. However, similar to Fig 2, as competitive asymmetry increased species loss increased (*α* decreased), and the decrease in *α* was more dramatic the larger the initial number of species. One potential mechanism behind this species-number dependent change in *α* is that larger values of *N* offer a wider sampling of the matrix elements *P*_*ik*_, and thus increase the likelihood that a single competitor dominates over many other weaker competitors and/or that ‘ultra weak’ competitors emerge.

Another potential link between the initial species number and the ensemble survival probability was system-size. To test this, we ran simulations across the same range of species number at the highest value of competitive asymmetry for two different system sizes (*L* = 100 and *L* = 200). For both system sizes, survival probability dropped as the initial number of species increased (Fig 4B), but the smaller system experienced a significantly faster drop, indicating that within this limited range of conditions and all else being equal smaller structured systems statistically retain fewer species in the long-time limit. We also examined the role of disorder in this context; consistent with our previous results disorder had a relatively minor effect as determined by 95% confidence intervals of MLE for the values of *α* (see S7 Fig for confidence intervals). Together, these results add support to the finding that disorder at a fixed structural scale is not a strong determinant of stable coexistence under localized competition, but that system size and the number of initially competing species each modulate survival probability and hence equilibrium biodiversity in this model.

## Discussion

In this work we simulated locally competitive interactions in a spatial LV framework to provide evidence that–within the given range of structural scales and competitive interaction parameters–*in silico* ecosystems that exist in structured environments and exhibit all-to-all (ATA) competition can avoid the competitive exclusion that is essentially guaranteed in spatially isotropic systems. Other well-known ecological ‘games’, such as rock-paper-scissors (RPS) and its higher species-number analogs [77,89], are also examples of ATA competition. That is, values in the matrix *P* that produce stable intransitive (e.g. RPS) oscillations adhere to the same conditions, but are subject to additional constraints on their relative values. The RPS sub-class distinguishes itself by exhibiting two important features. First, unlike non-oscillatory systems that lie within ATA, oscillatory systems with spatial isotropy can exhibit stable representation of all species [43,75,90], albeit with each species in constant spatial flux. Second, our previous work showed that in a symmetric game of RPS [64], structure could have a destabilizing effect that ultimately led to extinction cascades ending with a single dominant species. Thus, while the vast majority of parameter combinations for *P*_*ik*_ likely yield systems that are stabilized by steric structure, RPS-like graphs present the possibility of being destabilized by steric structure. Given that virtually all natural environments present structural anisotropy, destabilization of oscillatory networks due to steric structure may contribute to answering why RPS-like networks are only rarely observed outside of the lab [21,91–93].

Similarly, systems in which species cooperate or have competitive alliances–neither of which adhere to the conditions of ATA competition–can, by virtue of the specific localization of each species, be stabilized by structure. For instance, consider the simplest, non-ATA graph for which this can be true: *A* and *B* compete, *B* and *C* compete, and *A* predates *C* (see S8A Fig). If in a structured environment species *C* is stably and spatially isolated from *A* by the arrangement of *B*, then all three species could stably coexist due to the effects of steric structure, even though an interspecies boundary between *A* and *C* is unstable regardless of structural parameters. The idea that specific spatial arrangements of species can be stable in a structured environment extends to other non-ATA graphs (e.g. S8B Fig), and is consistent with established findings that spatially structured communities maintain biodiversity by localizing interactions among community members [94–96]. This also supports the hypothesis that physical structure and positioning of species play a role in shaping ecological and evolutionary relationships. Thus assessing the interplay between physical structure, graph structure, and ecological dynamics is a rich area of inquiry, one in which structure plays a qualitatively important role.

For simplicity and ease-of-display we explored 2D systems in this work, however many natural systems are three dimensional. This work does not allow us to directly comment on what will happen in 3D systems, however: (i) the structure of an ecological graph and its attendant parameters as encoded by the interaction matrix are independent of dimensionality, (ii) the relationship between interface curvature and competitive asymmetry that underlies many of the results herein described has a natural extension into three dimensions, where the mean curvature of a 2D interspecies boundary in 3D space plays the analogous role as 1D curvature of a line interface in 2D space, and (iii) the measures herein described (e.g. dimensionless disorder, structural scale, connection density, survival probabilities, etc.) have natural extensions into 3D, meaning that direct comparisons can be made between 2D and 3D systems. Further, there are other physical and biological systems whose behavior is also significantly (and often similarly) affected by the presence of steric structure, in particular the pinning phenomena that here slow or halt genetic (i.e. species) coarsening play important roles in domain-wall stabilization in Ising-like systems due to pinning at random spatial impurities [97], pinning-induced transitions of super-cooled liquids into glassy states [98], arrest of lipid-bilayer domain coarsening in the presence of biopolymers that impose structure on the bilayer [99], and genotype-specific range expansion [100]. Likewise, other physical mechanisms, such as flow [101], have been found to slow or halt coarsening in phase-separating systems, and still other work focuses on flow [102,103] or chemical gradients [104] in structuring communities. Thus the impact of steric structure on ecological communities both relates to other physical and biophysical systems, and is one of a multitude physical mechanisms shaping communities in complex environments.

Finally, even within this 2D reductionist framework we wonder how robust are pinned competition interfaces to: (i) stochastic spatial fluctuations caused either by finite organism size or other forms of motility (besides diffusion), (ii) the addition of other spatial anisotropies like chemical gradients or flow, and (iii) tunable interaction strengths, such as with competition sensing [105,106] or phenotypic differentiation [107]? Whether the details of interspecies interactions matter [74] or not [108], the stabilizing effect of structure observed with this model is fundamentally related to how interspecies boundaries–which appear in many extensions of the LV model–interact with the boundary conditions imposed by structure. Hence we anticipate that qualitatively, these results extend to a wider class of locally interacting, boundary-forming models (e.g. incorporating Allee effects [64]). Ultimately, an understanding of the interplay between ecological relationships, environmental structure, and other physical factors (like flow or chemical gradients) paves the way toward rational design of structured environments that tune the range of competitive asymmetries and/or stochastic fluctuations that an environment can stably support, and shifts system dynamics and stability to favor particular species or interaction topologies.

## Methods

In the 2D simulation space, each species was seeded by randomly choosing 0.2% of all valid pixels in the simulation box and setting the concentration of that species to 1/*N* of the carrying capacity, where *N* is the number of species being simulated; each species was represented by its own field matrix. Steric pillars with the specified radius *R*, spacing Δ*x*, and degree of disorder *δ* were placed within a simulation box whose side lengths were *L*. All reported length measures (*R*, *L*, Δ*x*) are in units of *λ*, with the computational pixel scale set so that $1.29\sqrt{D}=1.29\;\lambda =5$, i.e. pixels are smaller than the natural length scale and competition boundaries exist over scales of order 10 pixels. Microbial density that coincided with pillar locations was removed from the simulation. The bounding box and pillar edges were modeled with reflecting boundary conditions. At each simulated time step (Δ*t* = 0.01*t*, with *t* in natural units of inverse growth rate), populations diffused via a symmetric and conservative Gaussian convolution filter with standard deviation set by the diffusion coefficient, $\sigma =\sqrt{4D\Delta t}$. After the diffusion step, changes in population density (growth and death) were calculated using the equations given in the main text, and used to update the density of each species matrix according to a forward Euler scheme. In combination with the small dimensionless time-step and concentration-conserving convolution filter, hard upper and lower bounds (1 and 0 in units of carrying capacity, respectively) were enforced on each species field to ensure numerical stability of simulations; populations densities outside this range were set to 1 and 0, respectively. We monitored a subset of the simulations and found that simulations were sufficiently stable that those hard limits were never encountered. For each set of simulations with fixed structural scale (Δ*x*/*R*) and competitive asymmetry (Δ*P*/⟨*P*⟩), 30 to 50 independently initialized replicates were simulated for 5000 doubling times or until equilibrium was reached (whichever came first). Equilibrium was defined by the spatially averaged change in all species matrices falling below 0.001 of the carrying capacity between time steps. Mean population abundances of the simulation were recorded at an interval of 100 Δ*t* for the duration of the simulation. Extinction was defined as the mean population density of any species dropping below a threshold value of ((2*R*)^2^ − *πR*^2^)/4*A*, where *R* is the pillar radius and *A* the area of lattice points not obstructed by pillars. This non-zero threshold accounts for surviving populations ‘trapped’ between a pillar and the corner of the simulation box and therefore not in contact with the rest of the simulation; this threshold value lies well below the minimum domain size set by the local Delaunay triangulation of steric objects.

To create a controlled level of pillar-position disorder, each pillar center was displaced from a triangular lattice in a random direction by an amount drawn from a uniform random distribution whose width (*w*) was characterized by the dimensionless parameter defined as *δ* = 2*w*/(Δ*x* − *R*). The competition parameters between all species were generated by choosing the mean strength of competition ⟨*P*⟩ = 0.25 and then sampling a uniform random distribution of full width Δ*P* about that mean for each directed interaction, subject to the constraint that Δ*P*/2 < ⟨*P*⟩, which ensures that all random samplings adhere to the constraints of ATA competition.

Fitting to the modified binomial distribution was performed using maximum-likelihood estimation with all 1,000 simulations for each set of conditions; reported uncertainties for *α* are 95% confidence intervals.

## Supporting information

S1 Fig The pairwise distance distribution as a function of the disorder parameter *δ*. The distribution of separations between any two steric pillars does not depend on the size of the space in which those objects exist, assuming that the density of those objects is held fixed. For a perfect triangular lattice (*δ* = 0), that distribution is a series of delta-functions (purple dots). Adding structural disorder (*δ* > 0) makes the distributions continuous, with increasing degrees of disorder ultimately approaching the distribution expected for randomly placed objects at a fixed density (black line). As *δ* increases, the arrangement of steric pillars transitions smoothly from a triangular lattice to a random arrangement–in this work, we explored 0 ≤ *δ* ≤ 1.
(PDF)

S2 Fig Many species can form a junction around a single steric object. Here a simulation is contrived via initial conditions to have 12 species stably coexist, each making contact with the same steric object (gray circle in the center). The outer edge is also circular which permits stable coexistence of multiple species in a single open space. This figure demonstrates that, for a sufficiently large steric object as compared to the width of the competition interface, any number of species can ‘share’ a boundary with an object. However, in an environment with many steric objects in proximity, the local Voronoi neighborhood limits the maximum number of species that can exist around an object, usually to 3 or 4.
(PDF)

S3 Fig Survival distributions for a smaller system size. The type and format of data herein presented are the same as in Fig 3B–the only difference is that here the simulation box is half the linear size (1/4 the area). Simulations were performed to measure the probability distribution for the number of surviving species under four conditions (1,000 each): high and low competitive asymmetry and high and low structural disorder. The structural scale was held fixed. We used a maximum likelihood estimator (MLE) to measure the ensemble average survival probability (*α*) under those four conditions. Without competitive asymmetry (red and green X’s), the number of surviving species was heavily weighted toward the maximum possible number (8). With high competitive asymmetry, the probability distributions spread across all possible numbers of species with relatively little distinction between ordered and disordered systems (cyan and purple X’s). In all instances, the corresponding MLE fits are shown as solid lines. These results support the hypothesis that, all else being equal, smaller structured environments maintain fewer coexisting species in the long-time limit as compared to larger systems. In all simulations *L* = 75, ⟨*P*⟩ = 0.25, and Δ*x*/*R* = 3.5.
(PDF)

S4 Fig The effects of competitive asymmetry on mean coexistence are independent of structural disorder. To determine if, for the given parameter ranges, the impact of steric disorder on the mean number of surviving species was independent of the effect of competitive asymmetry, we correlated each row of Fig 3D with every other row of the same figure (55 unique correlations). All of those correlations were greater than 0.928, and the mean of all of those correlations was 0.969, meaning that over the range of steric disorder 0 ≤ *δ* ≤ 1 (and other fixed parameters) the relationship between mean number of surviving species and competitive asymmetry was approximately independent of disorder.
(PDF)

S5 Fig Connection-density statistics of competition boundaries. Using the same 4,000 simulations from Fig 3, we examined the density of interspecies boundaries under those four conditions. **(A)** A sample image of 8 competing species in equilibrium in a disordered steric environment. Species establish spatial domains (solid colors) with dark boundaries between those domains where active competition takes place. **(B)** Across all 4,000 simulations (though here for the same image as A), custom image analysis software examined the positions of each pillar (white) and the corresponding Voronoi tessellation (magenta tessellation) that indicates which pillars are Voronoi nearest neighbors. Image analysis algorithms segmented the interspecies boundaries between pillars and classified them according to how many pillars a boundary connected (blue = 2, green = 3, orange = 4). Approximately 2% of all connections were not Voronoi nearest neighbors (data not shown), and thus these connections were not used in this analysis, as were boundaries that made contact with the edge of the simulation box (gray). **(C)** For each set of conditions, here shown as four colors (same colors as in Fig 3B), we calculated the number of connections made by competition boundaries between Voronoi nearest neighbors as compared to the maximum possible number of boundaries (boundaries between all Voronoi nearest neighbors)–in the text called ‘connection density’. While there is a notable difference between ordered and disordered connection density when competition is balanced (red and green), the salient difference is between balanced (red/green) and asymmetric (cyan/purple) competition. Systems with asymmetric competition establish significantly fewer boundaries connecting nearest-neighbor steric objects, consistent with their higher abundance variability, and thus there is effectively less competition (i.e. fewer interfaces) in asymmetric systems.
(PDF)

S6 Fig Local statistics of Voronoi tessellation as a function of the structural disorder. **(left)** Distributions of distances between Voronoi nearest neighbors. In a perfect triangular lattice this distribution is a delta-function (black dashed line). As disorder increases the distribution of nearest neighbor distances spreads out and a significant fraction of neighbors are found closer together than in a triangular lattice of the same overall density. The vast majority of interspecies boundaries are between Voronoi nearest neighbors. Thus widening of the distribution impacts whether a particular interspecies boundary is stable, because the maximum curvature and hence maximum competitive asymmetry that is stable at a boundary is set by the distance between the objects that the competitive boundary connects. The inset shows an example of a Voronoi neighborhood and the local distances being measured (dark blue lines). **(right)** We examined hypothetical domains defined by the convex polygon of nearest neighbors around a steric object (see inset)–this defines a consistent region inside of which a competitor could stably exist (many other polygons could also be drawn). Across a large number of such polygon domains, we measured the distribution of longest edge lengths as a function of structural disorder. If the longest edge is less than the triangular lattice constant with the same overall density, then this domain is guaranteed to be more stable to competitive asymmetry than an ordered polygon at the same steric density. As disorder increases, the fraction of all such polygons that meet this more stringent stability condition increases (to the left of the black, dashed vertical line), supporting the hypothesis that disorder should have a stabilizing effect when competition is asymmetric, contingent on there being a sufficiently large area and hence sufficient opportunities for such domains to exist.
(PDF)

S7 Fig Dependence of ensemble survival probability on system size, disorder, and initial species number. Same exact data as shown in Fig 4B in the text, separated by value of *δ* and shown with 95% confidence intervals (dashed lines) determined by maximum-likelihood estimation. Variations caused by differences in *δ* are not significant, but variations caused by a difference in system size are significant.
(PDF)

S8 Fig Specific spatial arrangements of non-ATA competition can also be stabilized by steric structure. **(A)** The simplest example of a non-ATA graph (top) for which species abundances are stable in a structured environment–a single matrix element (arrow from *C* to *A*) breaks the ATA competition condition that all values of *P*_*ik*_ > 0. The schematic (bottom) shows an arrangement of the three species that is stable in a structured environment, even though an interspecies boundary between *A* and *C* is not stable in any structured environment; this is the only arrangement that can stably support all three species for the graph shown. **(B)** A second, more complex example where four species can be arranged to yield stable abundances of all species, regardless of the interaction between species *A* and *C* (no connection shown). (bottom) The schematic shows three possible arrangements of the four species that could allow *A* and *C* to coexist regardless of their interaction. These schematics assume that the competition boundaries (black lines) are stable for some set of interaction and structural parameters.
(PDF)
