# Supplementary material for: Structured environments foster competitor coexistence by manipulating interspecies interfaces
Source: PLoS Comput Biol. 2021 Jan 7;17(1):e1007762. doi: 10.1371/journal.pcbi.1007762 (PMC7790539; doi:10.1371/journal.pcbi.1007762)
Supplement: S1 Fig — The distribution of separations between any two steric pillars does not depend on the size of the space in which those objects exist, assuming that the density of those objects is held fixed. For a perfect triangular lattice (δ = 0), that distribution is a series of delta-functions (purple dots). Adding structural disorder (δ > 0) makes the distributions continuous, with increasing degrees of disorder ultimately approaching the distribution expected for randomly placed objects at a fixed density (black line). As δ increases, the arrangement of steric pillars transitions smoothly from a triangular lattice to a random arrangement–in this work, we explored 0 ≤ δ ≤ 1. (PDF) [file pcbi.1007762.s001.pdf]

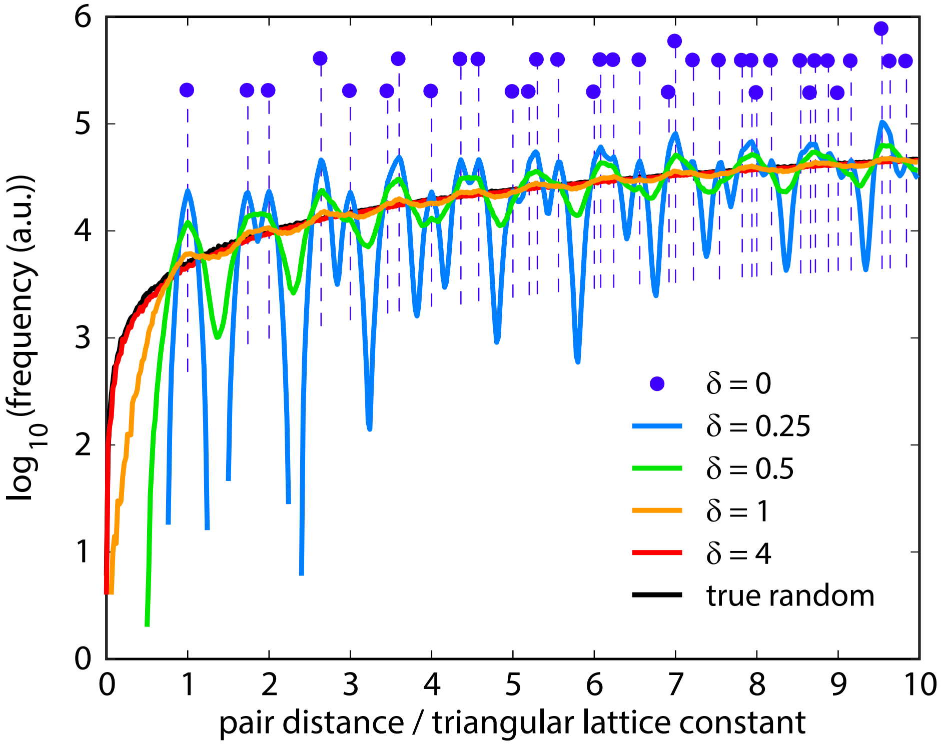

S1 Fig. The pairwise distance distribution as a function of the disorder parameter δ. The distribution of separations between any two steric pillars does not depend on the size of the space in which those objects exist, assuming that the density of those objects is held fixed. For a perfect triangular lattice ($\delta = 0$), that distribution is a series of delta-functions (purple dots). Adding structural disorder ($\delta > 0$) makes the distributions continuous, with increasing degrees of disorder ultimately approaching the distribution expected for randomly placed objects at a fixed density (black line). As δ increases, the arrangement of steric pillars transitions smoothly from a triangular lattice to a random arrangement – in this work, we explored $0 \leq \delta \leq 1$.
