# Supplementary material for: Structured environments foster competitor coexistence by manipulating interspecies interfaces
Source: PLoS Comput Biol. 2021 Jan 7;17(1):e1007762. doi: 10.1371/journal.pcbi.1007762 (PMC7790539; doi:10.1371/journal.pcbi.1007762)
Supplement: S2 Fig — Here a simulation is contrived via initial conditions to have 12 species stably coexist, each making contact with the same steric object (gray circle in the center). The outer edge is also circular which permits stable coexistence of multiple species in a single open space. This figure demonstrates that, for a sufficiently large steric object as compared to the width of the competition interface, any number of species can ‘share’ a boundary with an object. However, in an environment with many steric objects in proximity, the local Voronoi neighborhood limits the maximum number of species that can exist around an object, usually to 3 or 4. (PDF) [file pcbi.1007762.s002.pdf]

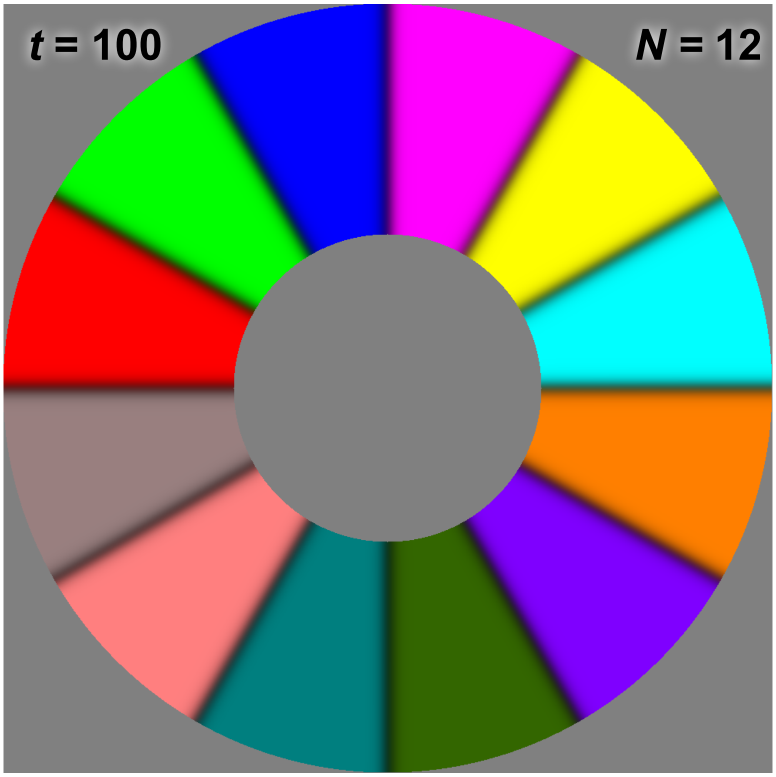


**S2 Fig. Many species can form a junction around a single steric object**. Here a simulation is contrived via initial conditions to have 12 species stably coexist, each making contact with the same steric object (gray circle in the center). The outer edge is also circular which permits stable coexistence of multiple species in a single open space. This figure demonstrates that, for a sufficiently large steric object as compared to the width of the competition interface, any number of species can 'share' a boundary with an object. However, in an environment with many steric objects in proximity, the local Voronoi neighborhood limits the maximum number of species that can exist around an object, usually to 3 or 4.
