# Supplementary material for: Structured environments foster competitor coexistence by manipulating interspecies interfaces
Source: PLoS Comput Biol. 2021 Jan 7;17(1):e1007762. doi: 10.1371/journal.pcbi.1007762 (PMC7790539; doi:10.1371/journal.pcbi.1007762)
Supplement: S3 Fig — The type and format of data herein presented are the same as in Fig 3B–the only difference is that here the simulation box is half the linear size (1/4 the area). Simulations were performed to measure the probability distribution for the number of surviving species under four conditions (1,000 each): high and low competitive asymmetry and high and low structural disorder. The structural scale was held fixed. We used a maximum likelihood estimator (MLE) to measure the ensemble average survival probability (α) under those four conditions. Without competitive asymmetry (red and green X’s), the number of surviving species was heavily weighted toward the maximum possible number (8). With high competitive asymmetry, the probability distributions spread across all possible numbers of species with relatively little distinction between ordered and disordered systems (cyan and purple X’s). In all instances, the corresponding MLE fits are shown as solid lines. These results support the hypothesis that, all else being equal, smaller structured environments maintain fewer coexisting species in the long-time limit as compared to larger systems. In all simulations L = 75, ⟨P⟩ = 0.25, and Δx/R = 3.5. (PDF) [file pcbi.1007762.s003.pdf]

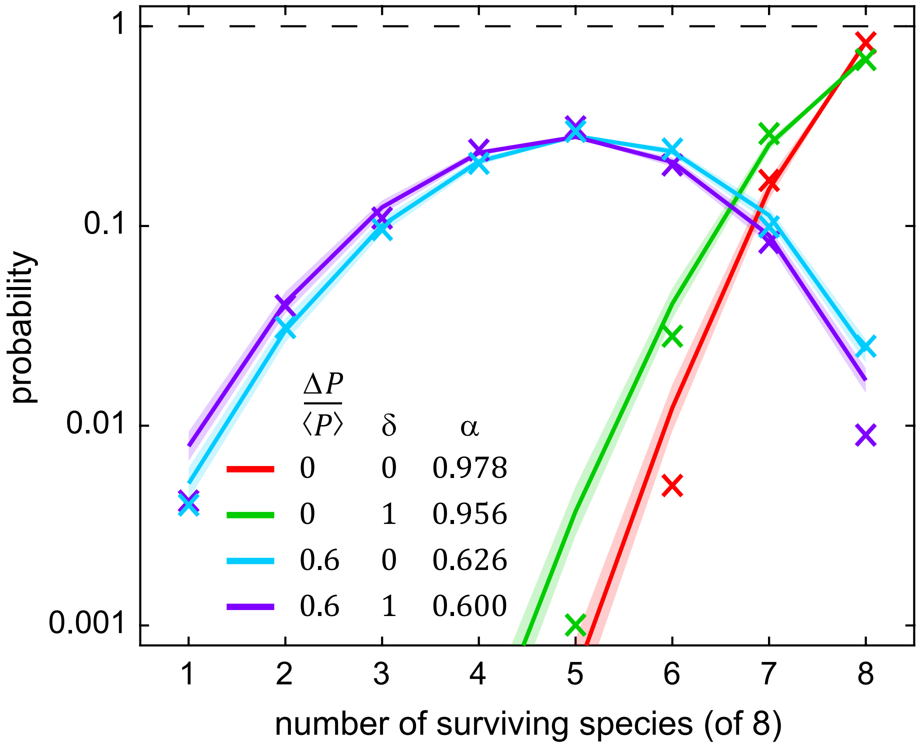

S3 Fig. Survival distributions for a smaller system size. The type and format of data herein presented are the same as in Fig 3B – the only difference is that here the simulation box is half the linear size (1/4 the area). Simulations were performed to measure the probability distribution for the number of surviving species under four conditions (1,000 each): high and low competitive asymmetry and high and low structural disorder. The structural scale was held fixed. We used a maximum likelihood estimator (MLE) to measure the ensemble average survival probability (α) under those four conditions. Without competitive asymmetry (red and green X's), the number of surviving species was heavily weighted toward the maximum possible number (8). With high competitive asymmetry, the probability distributions spread across all possible numbers of species with relatively little distinction between ordered and disordered systems (cyan and purple X's). In all instances, the corresponding MLE fits are shown as solid lines. These results support the hypothesis that, all else being equal, smaller structured environments maintain fewer coexisting species in the long-time limit as compared to larger systems. In all simulations $L = 75$, $\langle P \rangle = 0.25$, and $\Delta x/R = 3.5$.
