# Supplementary material for: Structured environments foster competitor coexistence by manipulating interspecies interfaces
Source: PLoS Comput Biol. 2021 Jan 7;17(1):e1007762. doi: 10.1371/journal.pcbi.1007762 (PMC7790539; doi:10.1371/journal.pcbi.1007762)
Supplement: S4 Fig — To determine if, for the given parameter ranges, the impact of steric disorder on the mean number of surviving species was independent of the effect of competitive asymmetry, we correlated each row of Fig 3D with every other row of the same figure (55 unique correlations). All of those correlations were greater than 0.928, and the mean of all of those correlations was 0.969, meaning that over the range of steric disorder 0 ≤ δ ≤ 1 (and other fixed parameters) the relationship between mean number of surviving species and competitive asymmetry was approximately independent of disorder. (PDF) [file pcbi.1007762.s004.pdf]

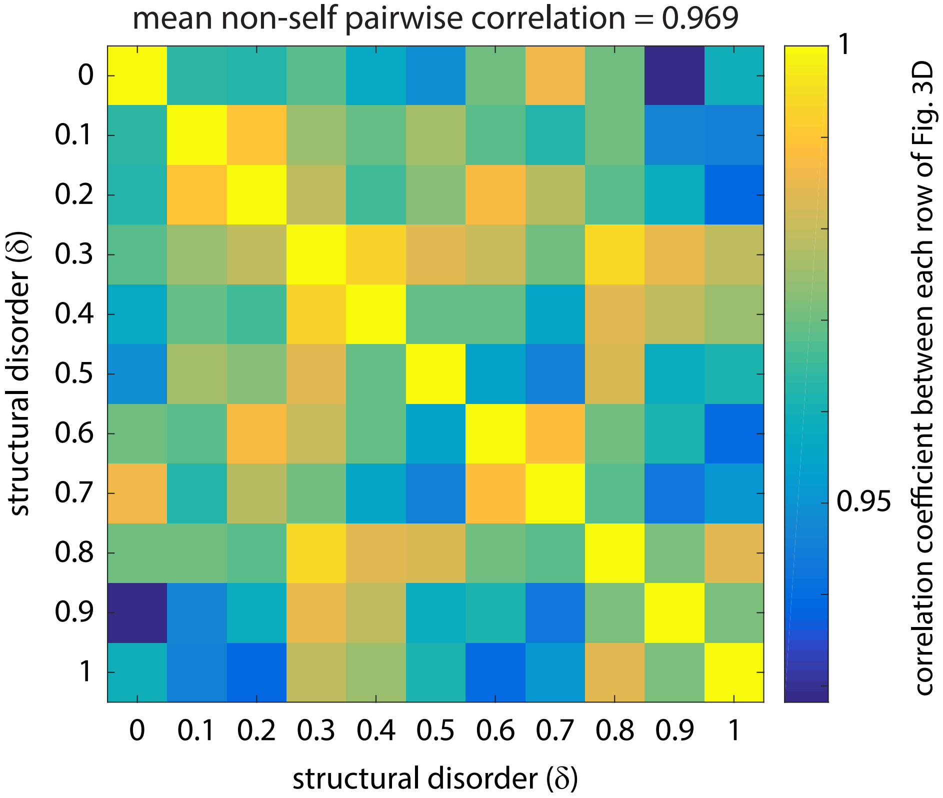

S4 Fig. The effects of competitive asymmetry on mean coexistence are independent of structural disorder. To determine if, for the given parameter ranges, the impact of steric disorder on the mean number of surviving species was independent of the effect of competitive asymmetry, we correlated each row of Fig 3D with every other row of the same figure (55 unique correlations). All of those correlations were greater than 0.928, and the mean of all of those correlations was 0.969, meaning that over the range of steric disorder $0 \leq \delta \leq 1$ (and other fixed parameters) the relationship between mean number of surviving species and competitive asymmetry was approximately independent of disorder.
