# Supplementary material for: Structured environments foster competitor coexistence by manipulating interspecies interfaces
Source: PLoS Comput Biol. 2021 Jan 7;17(1):e1007762. doi: 10.1371/journal.pcbi.1007762 (PMC7790539; doi:10.1371/journal.pcbi.1007762)
Supplement: S5 Fig — Using the same 4,000 simulations from Fig 3, we examined the density of interspecies boundaries under those four conditions. (A) A sample image of 8 competing species in equilibrium in a disordered steric environment. Species establish spatial domains (solid colors) with dark boundaries between those domains where active competition takes place. (B) Across all 4,000 simulations (though here for the same image as A), custom image analysis software examined the positions of each pillar (white) and the corresponding Voronoi tessellation (magenta tessellation) that indicates which pillars are Voronoi nearest neighbors. Image analysis algorithms segmented the interspecies boundaries between pillars and classified them according to how many pillars a boundary connected (blue = 2, green = 3, orange = 4). Approximately 2% of all connections were not Voronoi nearest neighbors (data not shown), and thus these connections were not used in this analysis, as were boundaries that made contact with the edge of the simulation box (gray). (C) For each set of conditions, here shown as four colors (same colors as in Fig 3B), we calculated the number of connections made by competition boundaries between Voronoi nearest neighbors as compared to the maximum possible number of boundaries (boundaries between all Voronoi nearest neighbors)–in the text called ‘connection density’. While there is a notable difference between ordered and disordered connection density when competition is balanced (red and green), the salient difference is between balanced (red/green) and asymmetric (cyan/purple) competition. Systems with asymmetric competition establish significantly fewer boundaries connecting nearest-neighbor steric objects, consistent with their higher abundance variability, and thus there is effectively less competition (i.e. fewer interfaces) in asymmetric systems. (PDF) [file pcbi.1007762.s005.pdf]

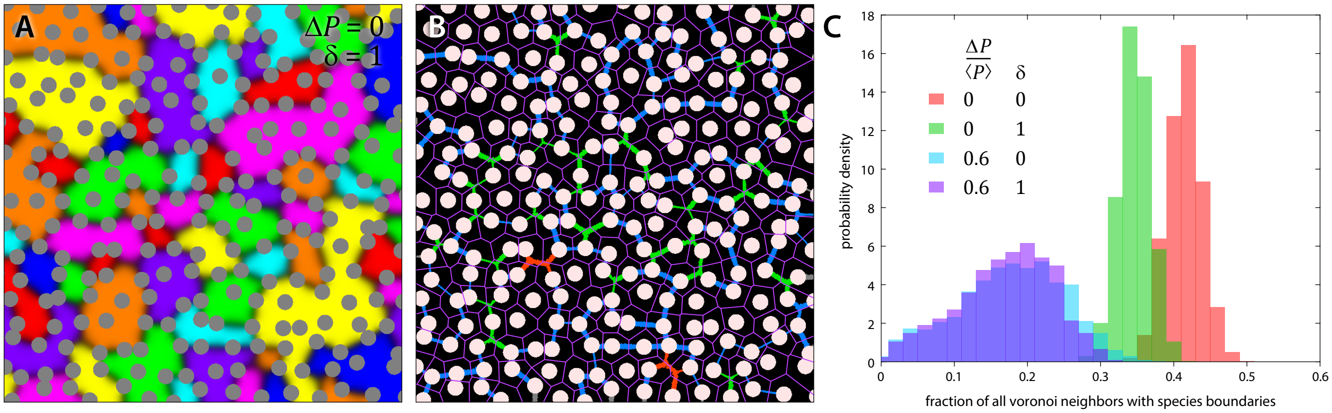

S5 Fig. Connection-density statistics of competition boundaries. Using the same 4,000 simulations from Fig 3, we examined the density of interspecies boundaries under those four conditions. **(A)** A sample image of 8 competing species in equilibrium in a disordered steric environment. Species establish spatial domains (solid colors) with dark boundaries between those domains where active competition takes place. **(B)** Across all 4,000 simulations (though here for the same image as A), custom image analysis software examined the positions of each pillar (white) and the corresponding Voronoi tessellation (magenta tessellation) that indicates which pillars are Voronoi nearest neighbors. Image analysis algorithms segmented the interspecies boundaries between pillars and classified them according to how many pillars a boundary connected (blue = 2, green = 3, orange = 4). Approximately 2% of all connections were not Voronoi nearest neighbors, and thus these connections were not used in this analysis, as were boundaries that made contact with the edge of the simulation box (gray). **(C)** For each set of conditions, here shown as four colors (same colors as in Fig 3B), we calculated the number of connections made by competition boundaries between Voronoi nearest neighbors as compared to the maximum possible number of boundaries (boundaries between all Voronoi nearest neighbors) – in the text called 'connection density'. While there is a notable difference between ordered and disordered connection density when competition is balanced (red and green), the salient difference is between balanced (red / green) and asymmetric (cyan / purple) competition. Systems with asymmetric competition established significantly fewer boundaries connecting nearest-neighbor steric objects, consistent with their higher abundance variability, and thus there is effectively less competition (i.e. fewer interfaces) in asymmetric systems.
