# Supplementary figures and images for: Structured environments foster competitor coexistence by manipulating interspecies interfaces
Source: PLoS Comput Biol. 2021 Jan 7;17(1):e1007762. doi: 10.1371/journal.pcbi.1007762 (PMC7790539; doi:10.1371/journal.pcbi.1007762)

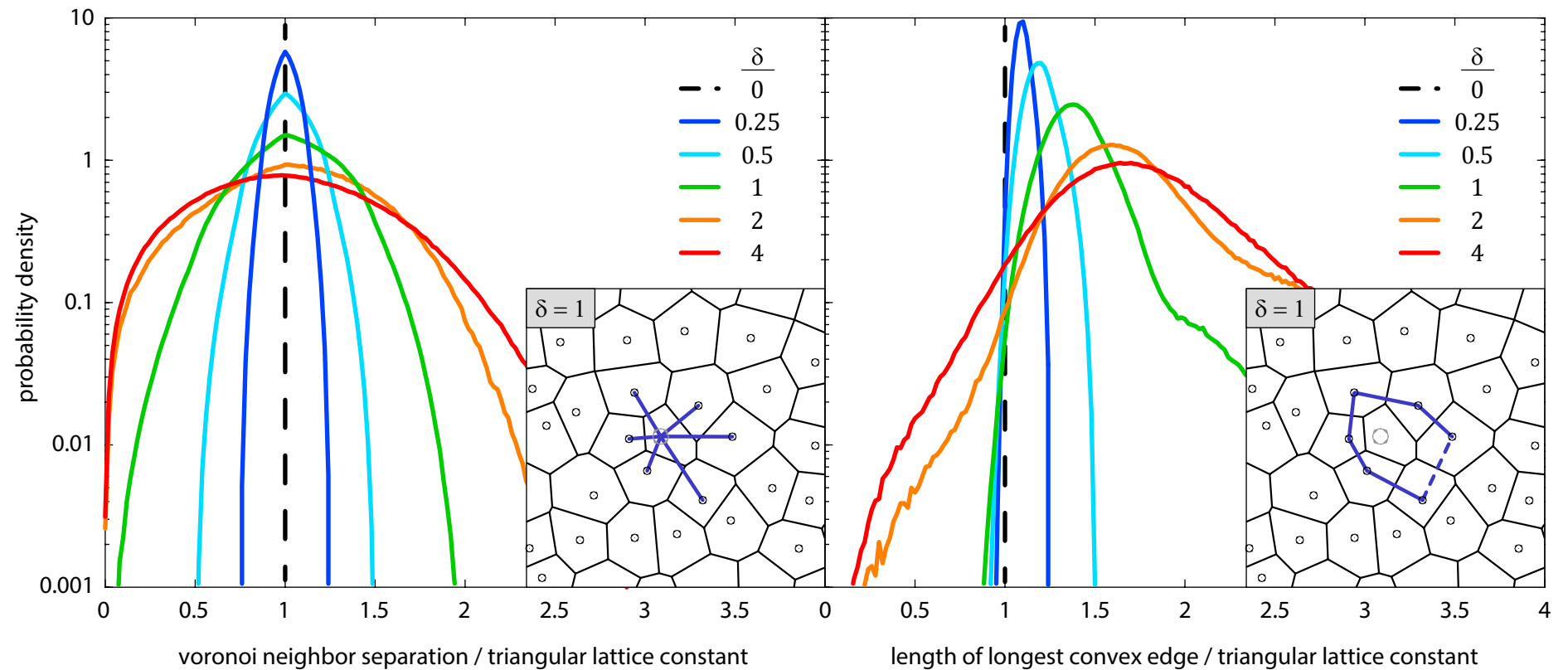

Supplement: S6 Fig — (left) Distributions of distances between Voronoi nearest neighbors. In a perfect triangular lattice this distribution is a delta-function (black dashed line). As disorder increases the distribution of nearest neighbor distances spreads out and a significant fraction of neighbors are found closer together than in a triangular lattice of the same overall density. The vast majority of interspecies boundaries are between Voronoi nearest neighbors. Thus widening of the distribution impacts whether a particular interspecies boundary is stable, because the maximum curvature and hence maximum competitive asymmetry that is stable at a boundary is set by the distance between the objects that the competitive boundary connects. The inset shows an example of a Voronoi neighborhood and the local distances being measured (dark blue lines). (right) We examined hypothetical domains defined by the convex polygon of nearest neighbors around a steric object (see inset)–this defines a consistent region inside of which a competitor could stably exist (many other polygons could also be drawn). Across a large number of such polygon domains, we measured the distribution of longest edge lengths as a function of structural disorder. If the longest edge is less than the triangular lattice constant with the same overall density, then this domain is guaranteed to be more stable to competitive asymmetry than an ordered polygon at the same steric density. As disorder increases, the fraction of all such polygons that meet this more stringent stability condition increases (to the left of the black, dashed vertical line), supporting the hypothesis that disorder should have a stabilizing effect when competition is asymmetric, contingent on there being a sufficiently large area and hence sufficient opportunities for such domains to exist. (PDF) [file pcbi.1007762.s006.pdf]
