# Supplementary material for: Structured environments foster competitor coexistence by manipulating interspecies interfaces
Source: PLoS Comput Biol. 2021 Jan 7;17(1):e1007762. doi: 10.1371/journal.pcbi.1007762 (PMC7790539; doi:10.1371/journal.pcbi.1007762)
Supplement: S7 Fig — Same exact data as shown in Fig 4B in the text, separated by value of δ and shown with 95% confidence intervals (dashed lines) determined by maximum-likelihood estimation. Variations caused by differences in δ are not significant, but variations caused by a difference in system size are significant. (PDF) [file pcbi.1007762.s007.pdf]

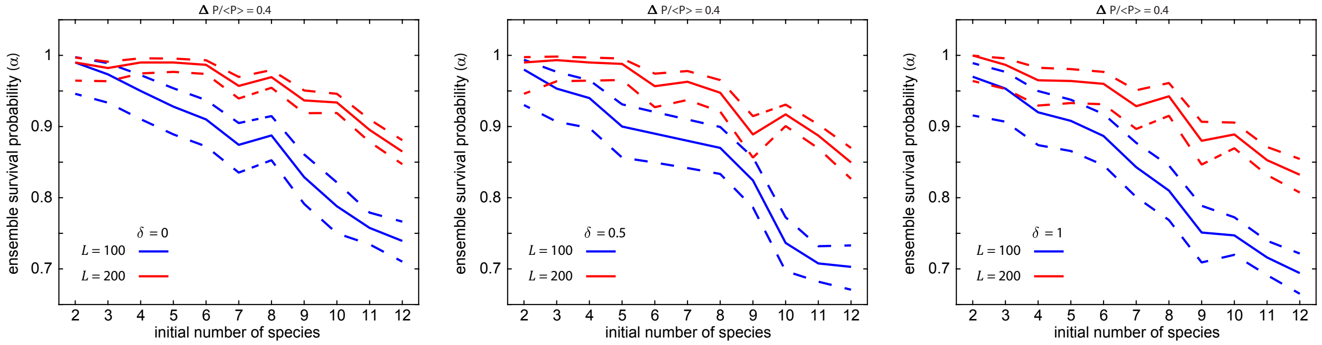

S7 Fig. Dependence of ensemble survival probability on system size, disorder, and initial species number. Same exact data as shown in Fig 4B in the text, separated by value of δ and shown with 95% confidence intervals (dashed lines) determined by maximum-likelihood estimation. Variations caused by differences in δ are not significant, whereas variations caused by a difference in system size are significant.
