# Supplementary material for: Structured environments foster competitor coexistence by manipulating interspecies interfaces
Source: PLoS Comput Biol. 2021 Jan 7;17(1):e1007762. doi: 10.1371/journal.pcbi.1007762 (PMC7790539; doi:10.1371/journal.pcbi.1007762)
Supplement: S8 Fig — (A) The simplest example of a non-ATA graph (top) for which species abundances are stable in a structured environment–a single matrix element (arrow from C to A) breaks the ATA competition condition that all values of Pik > 0. The schematic (bottom) shows an arrangement of the three species that is stable in a structured environment, even though an interspecies boundary between A and C is not stable in any structured environment; this is the only arrangement that can stably support all three species for the graph shown. (B) A second, more complex example where four species can be arranged to yield stable abundances of all species, regardless of the interaction between species A and C (no connection shown). (bottom) The schematic shows three possible arrangements of the four species that could allow A and C to coexist regardless of their interaction. These schematics assume that the competition boundaries (black lines) are stable for some set of interaction and structural parameters. (PDF) [file pcbi.1007762.s008.pdf]

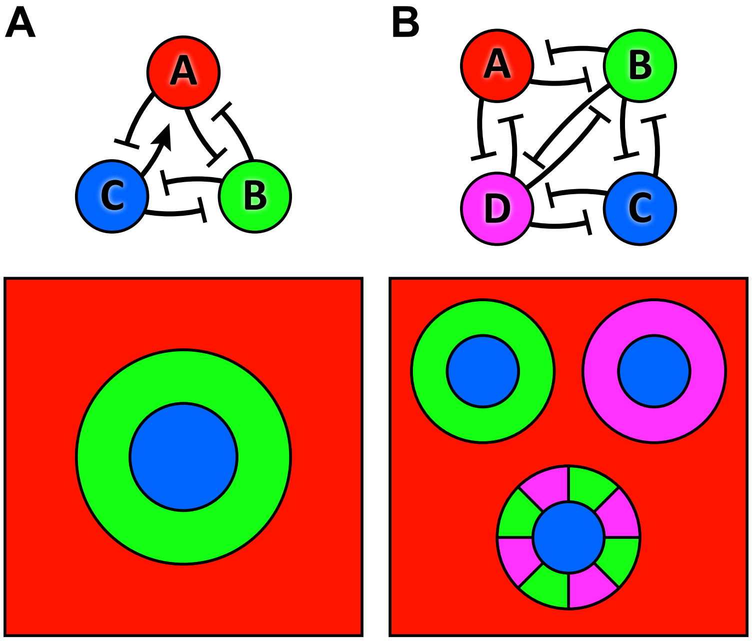

S8 Fig. Specific spatial arrangements of non-ATA competition can also be stabilized by steric structure. **(A)** The simplest example of a non-ATA graph (top) for which species abundances are stable in a structured environment – a single matrix element (arrow from C to A) breaks the ATA competition condition that all values of $P_{ik} > 0$. The schematic (bottom) shows an arrangement of the three species that is stable in a structured environment, even though an interspecies boundary between A and C is not stable in any structured environment. **(B)** A second, more complex example where four species can be arranged to yield stable abundances of all species, regardless of the interaction between species A and C (no connection shown). (bottom) The schematic shows three possible arrangements of the four species that could allow A and C to coexist regardless of their interaction. These schematics assume that the competition boundaries (black lines) are stable for some set of interaction and structural parameters.
